# A Novel Combination of Genes Causing Temperature-Sensitive Hybrid Weakness in Rice

**DOI:** 10.3389/fpls.2022.908000

**Published:** 2022-06-28

**Authors:** Than Kutay Soe, Mai Kunieda, Hidehiko Sunohara, Yoshiaki Inukai, Vincent Pamugas Reyes, Shunsaku Nishiuchi, Kazuyuki Doi

**Affiliations:** ^1^Laboratory of Information Sciences in Agricultural Lands, Graduate School of Bioagricultural Sciences, Nagoya University, Nagoya, Japan; ^2^Department of Botany, University of Yangon, Yangon, Myanmar; ^3^Environmental Control Center Co., Ltd., Hachioji, Japan; ^4^International Center for Research and Education in Agriculture, Nagoya University, Nagoya, Japan

**Keywords:** reproductive isolation, hybrid weakness, genotyping-by-sequencing (GBS), rice, QTL

## Abstract

Reproductive isolation is an obstacle for plant breeding when a distant cross is demanded. It can be divided into two main types based on different growth stages: prezygotic isolation and postzygotic isolation. The hybrid weakness, which is a type of postzygotic isolation, can become a problem in crop breeding. In order to overcome reproductive isolation, it is necessary to elucidate its mechanism. In this study, genetic analysis for low temperature-dependent hybrid weakness was conducted in a rice F_2_ population derived from Taichung 65 (T65, Japonica) and Lijiangxintuanheigu (LTH, Japonica). The weak and severe weak plants in F_2_ showed shorter culm length, late heading, reduced panicle number, decreased grain numbers per panicle, and impaired root development in the field. Our result also showed that hybrid weakness was affected by temperature. It was observed that 24°C enhanced hybrid weakness, whereas 34°C showed recovery from hybrid weakness. In terms of the morphology of embryos, no difference was observed. Therefore, hybrid weakness affects postembryonic development and is independent of embryogenesis. The genotypes of 126 F_2_ plants were determined through genotyping-by-sequencing and a linkage map consisting of 862 single nucleotide polymorphism markers was obtained. Two major quantitative trait loci (QTLs) were detected on chromosomes 1 [*hybrid weakness j 1* (*hwj1*)] and 11 [*hybrid weakness j 2* (*hwj2*)]. Further genotyping indicated that the hybrid weakness was due to an incompatible interaction between the T65 allele of *hwj1* and the LTH allele of *hwj2*. A large F_2_ populations consisting of 5,722 plants were used for fine mapping of *hwj1* and *hwj2*. The two loci, *hwj1* and *hwj2*, were mapped in regions of 65-kb on chromosome 1 and 145-kb on chromosome 11, respectively. For *hwj1*, the 65-kb region contained 11 predicted genes, while in the *hwj2* region, 22 predicted genes were identified, two of which are disease resistance-related genes. The identified genes along these regions serve as preliminary information on the molecular networks associated with hybrid weakness in rice.

## Introduction

In plant breeding, the development of new varieties requires expansion of genetic diversity, subsequent selection, and uniformization. The expansion of genetic diversity is the initial and one of the important steps to exploiting the diversity of genes available for breeding. For this purpose, several methods such as introduction, crossing, mutagenesis, and transgenic have been used. Among these methods, the crossing is the most frequently used. The genetic diversity of materials used for crossing, especially of distant relatives, often cause abnormality of the hybrids and progeny, called the reproductive barrier. To facilitate the use of various genetic resources and to transfer useful genes to new varieties, an intermediate set of materials that breeders can use as the starting materials, which is known as pre-breeding (Kumar and Shukla, 2014), is proposed.

In rice, crosses between different genomic species of the genus *Oryza* are often very difficult due to the crossing barriers and abnormal chromosome pairing in meiosis (Vaughan et al., 2003). There are several reports on the successful construction of pre-breeding materials (introgression lines) obtained from the crosses between *Oryza sativa* and other AA-genome species (e.g., Yoshimura et al., 2010). In addition, *O. sativa* has been classified into Japonica and Indica, and crosses between this variety group are also performed (e.g., Kubo et al., 2002). The reproductive isolation was observed in these cross combinations (e.g., Kubo and Yoshimura, 2002). Therefore, reproductive isolation generally becomes a major obstacle in plant breeding.

Reproductive barriers are highly related to the genetic differentiation of populations within and between species (Coyne and Orr, 2004; Rieseberg and Willis, 2007). Based on the stage of growth, these reproductive barriers can be classified as prezygotic isolation and postzygotic isolation. Prezygotic isolation is a common phenomenon that prevents both an inter and an intra-specific crossing. This type of reproductive isolation is due to various factors such as geographical isolation, the difference in flowering time, pollinator specificity, incompatibility in pollen tube growth (Stebbins, 1950). In contrast, postzygotic reproductive isolation happens after the zygotes or hybrids are developed. This type of reproductive isolation often leads to embryonic lethality, seed inviability, weakness, and sterility. One of the fundamental theories on the mechanism of postzygotic isolation was explained by the Bateson–Dobzhansky–Muller (BDM) model (Dobzhansky, 1937; Muller, 1942; Coyne and Orr, 2004). This model postulates that the deleterious interaction of two or more genes, derived from different species or populations, causes postzygotic isolation.

Hybrid weakness refers to the phenomenon in which the hybrid, derived from two normal parents, shows defective development such as necrotic leaves, small stature, or poor growth (Bomblies and Weigel, 2007). This phenomenon has been reported in many other plant species, including *Arabidopsis thaliana* (Bomblies et al., 2007), *Phaseolus vulgaris* (Shii et al., 1980), interspecific crosses among *Gossypium* (Lee, 1981), interspecific crosses among *Nicotiana* (Tezuka et al., 2007), and interspecific crosses among *Capsicum* (Shiragaki et al., 2021).

Hybrid weakness in rice is frequently observed (Koide et al., 2008). To date, several studies have identified genes that are associated with hybrid weakness in rice. The very first study of hybrid weakness genes, *L*_1_ and *L*_2_ were reported by Oka (1957). In most cases, hybrid weakness was controlled by the complementary interaction of unlinked loci, e.g., *Hwc1* and *Hwc2* (Ichitani et al., 2001, 2007; Kuboyama et al., 2009), *hwd1* and *hwd2* (Fukuoka et al., 1998), *hwe1* and *hwe2* (Kubo and Yoshimura, 2002), *hwg1* and *hwg2* (Fukuoka et al., 2005), *hwh1* and *hwh2* (Jiang et al., 2008), *hwi1* and *hwi2* (Chen et al., 2013, 2014), and *hbd2* and *hbd3* (Matsubara et al., 2007; Yamamoto et al., 2007).

Previous studies suggested that the over-activated immune responses might be an essential mechanism for triggering hybrid weakness of rice, *Arabidopsis*, and other plant species (Bomblies et al., 2007; Alcázar et al., 2009, 2010; Jeuken et al., 2009; Chen et al., 2013, 2014; Shiragaki et al., 2021). For example, in *Arabidopsis*, an autoimmune response was activated to cause hybrid weakness by gene interaction between two loci and at least one of them coded nucleotide-binding site leucine-rich repeat (NB-LRR) protein (Bomblies et al., 2007). NB-LRR genes are the most common class of disease resistance (R) genes in plants (Jones and Dangl, 2006). In rice, one of the interacting loci for hybrid weakness coded NB-LRR genes (Yamamoto et al., 2010). Chen et al. (2014) reported that the interaction of leucine-rich repeat receptor-like protein kinase (LRR-RLK) genes from wild rice and the subtilisin-like protease gene from Indica rice activated the autoimmune response resulted in hybrid weakness. Temperature is known to influence disease resistance to pathogens. A high temperature mostly inhibits disease resistance (e.g., Dropkin, 1969). Only a few genes for hybrid weakness in rice have been cloned (Yamamoto et al., 2010; Chen et al., 2014; Nadir et al., 2019). Therefore, the molecular mechanism of hybrid weakness in rice still needs to be elucidated.

Clarifying the mechanisms of reproductive barriers such as hybrid weakness is important not only to understand speciation and evolution but also to overcome these barriers for crop breeding. Here, we report a new combination of loci causing temperature-sensitive hybrid weakness in rice. The genetic analysis of hybrid weakness was carried out using F_2_ populations derived from a cross between Taichung 65 (*Oryza sativa* Japonica) and Lijiangxintuanheigu (*O. sativa* Japonica). The two loci, *hwj1* and *hwj2*, associated with hybrid weakness, were fine mapped at the 65-kb region and 145-kb of chromosomes 1 and 11, respectively.

## Materials and Methods

### Plant Materials

**FIGURE 1:**
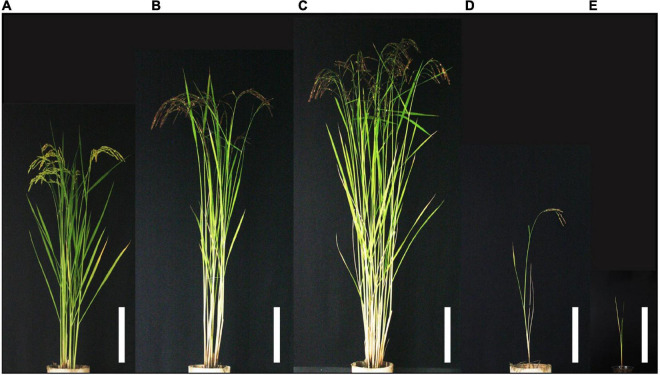
Morphology of F_2_ plants and parents at the reproductive stage. **(A)** T65, **(B)** Lijiangxintuanheigu (LTH), **(C)** normal plant, **(D)** weak plant, and **(E)** severe weak plant. Scale bars = 20 cm.

In this study, F_2_ populations derived from a cross between Taichung 65 (T65) and Lijiangxintuanheigu (LTH) were used (Figure 1). In summary, the seeds were pre-germinated in water at room temperature for 3 days and were sown. The 30-day-old seedlings were transplanted ten plants per row with 20 cm between the hill and 30 cm spacing between rows in Togo field, Nagoya University. The field experiments were done three times, in 2016, 2018, and 2019. In 2016, an F_2_ population consisting of 344 plants was grown and among them, 126 plants were used for genotyping by sequencing (GBS) and quantitative trait loci (QTL) analysis. In 2018 and 2019, large F_2_ populations consisting of 5,722 plants were used for fine mapping of *hwj1* and *hwj2*.

### Phenotypic Characterization of Hybrid Weakness

In 2016, culm length, days to heading, and panicle number per plant were recorded using 306 plants. In 2019, the number of grains per panicle, and filled and unfilled grain numbers in the main panicle were counted using ten mature plants for each genotype. Days to heading were recorded thrice in a week. Harvesting of the panicles of each plant was done 40-45 days after heading and samples were dried in a greenhouse. After the QTL analysis, all 344 F_2_ plants (in 2016) were genotyped using the DNA markers linked to the two causal loci, and trait values were compared among the genotypes using R software^1^.

### Genotyping-by-Sequencing and Quantitative Trait Loci Analysis

For DNA extraction, the leaf of each plant was taken three weeks after transplanting and was oven-dried at 52°C overnight. The DNA was extracted following a modified Dellaporta method (Dellaporta et al., 1983). The quality of extracted DNA was checked by electrophoresis on a 0.8% agarose gel in 0.5 × TBE buffer. For GBS materials, the QuantiFluor dsDNA System and Quantus fluorometer instrument (Promega, Madison, WI, United States) were used for the quantification of extracted DNA.

The genotypes of 126 F_2_ plants, from the F_2_ population consisting of 344 individual plants derived from the cross between T65 and LTH (in 2016), were determined following the protocol of Poland et al. (2012). Two combinations of the restriction enzymes, *Kpn*I-*Msp*I and *Pst*I-*Msp*I were used for the construction of the next generation sequencing (NGS) libraries. The NGS libraries were sequenced using Illumina MiSeq (Illumina, San Diego, CA, United States). The informatics was done following the pipeline used by Furuta et al. (2017).

The number of panicles of the 126 F_2_ plants was used as an indicator for QTL mapping for the population in 2016. The R/qtl package (Broman et al., 2003) was used for QTL analysis using “scanone” and “scantwo” functions for detecting 1-way and 2-way QTL. The LOD score significance threshold was calculated using 1,000 permutations.

### Fine Mapping of Causal Loci

In 2018 and 2019, a total of 5,722 plants from F_2_ populations were genotyped using InDel markers surrounding the two loci (Tables 1, 2). The informative plants were used for further fine mapping. To narrow down the candidate regions, cleaved amplified polymorphic sequence (CAPS) markers were designed and used (Supplementary Tables 1, 2).

**TABLE 1:** List of InDel markers for fine mapping of *hwj1* locus.

No.	Marker	Primer sequence	Position
1	IDM0144_f	ccccactgttcccaaaccgt	17,405,250
	IDM0144_r	tttaaccccctcaacttttcactct	
2	IDM0145_f	agcattggtatttacgtcgctaca	18,526,363
	IDM0145_r	actttgagcctcatttgttaccca	
3	IDM0147_f	gtcaccgttggtagccccac	18,863,466
	IDM0147_r	gcagcagtggagtgacacca	
4	IDSO0101_f	tgtgacacctgtttttatcttccgta	17,724,364
	IDSO0101_r	gcatgactggagatcttcgaatactta	
5	IDMSO0102_f	tccctcgatctcacggaatta	17,797,920
	IDMSO0102_r	cagggttagacgggaacgtg	
6	IDMSO0104_f	cgggtgaaataaccgggagt	18,272,024
	IDMSO0104_r	tgcatggtttaaccgggagt	
7	IDMSO0105_f	tctgcccttacctccctgaa	18,460,793
	IDMSO0105_r	tgtgctgtgtgtcgcgtatg	

**TABLE 2:** List of InDel markers for fine mapping of *hwj2* locus.

No.	Marker	Primer sequence	Position
1	IDM1104_f	tgttccagccaacgaacaca	22,425,872
	IDM1104_r	tctctacgacgcggcaaact	
2	IDM1106_f	cgtgggcaggaggtggaga	23,414,083
	IDM1106_r	tctgggccgtcagatctgct	
3	IDM1109_f	tgcaagattgtggcaagcgc	24,322,691
	IDM1109_r	agcaggcttgttggtgagtga	
4	IDMSO1101_f	actttccgatcgattagttgaca	23,488,904
	IDMSO1101_r	tccgtacgtaatctctagaaagaagaa	
5	IDMSO1102_f	tgttggtttgtgagggcagtc	23,693,281
	IDMSO1102_r	gcatctcgctgttggctgt	
6	IDMSO1103_f	cacgggggtcactggataag	23,748,995
	IDMSO1103_r	cacgccaaggtcatgcttc	
7	IDMSO1104_f	tgcctttcgagttcttcacca	24,085,843
	IDMSO1104_r	gcagctcaaagaaaaagaagagca	
8	IDMSO1105_f	gggacagatggtttttcttgtg	24,206,507
	IDMSO1105_r	tcagacaaccgttcacgaaa	
9	IDMSO1106_f	tcccattcctcatcactaactcc	24,210,608
	IDMSO1106_r	tgggttatgcatacgacagca	

### Temperature Sensitivity Test

To analyze the sensitivity to temperature, T65, LTH, and their F_2_ seeds were pre-germinated in water at room temperature for 3 days. The germinated seeds were sown on a plastic mesh in the water at 24 and 34°C in a growth chamber with continuous light. Ten days after sowing, the shoot length and root length of each plant were measured. Alongside these, the number of roots of each plant was also counted. To identify significant differences among genotypes, one-way analysis of variance (ANOVA) followed by Tukey’s HSD (honestly significant difference) test (*p* < 0.05) was performed through R software.

### Morphological and Histological Characterizations of Embryo

Prior to morphological and histological analyses of seeds, genotypes were determined using half seeds of the F_2_ populations. To observe the morphology and histology of the embryos, half seeds of all genotypes including parents were soaked at 24°C. The F_2_ seeds which possessed severe weak genotype (*aabb*), weak genotype (*aaBb*, *Aabb)*, and normal genotype (*AABB*), together with T65 and LTH, were soaked in water at 24°C. Embryos were observed 1 day after soaking under a digital microscope (Keyence VHX-600). The embryos were then fixed with 4% paraformaldehyde overnight at 4°C and embedded in SCEM medium (SECTION-LAB, Hiroshima, Japan). The cut surface was covered with an adhesive film (Cryofilm type IIC9, SECTION-LAB, Hiroshima, Japan) and frozen sections (20 μm thickness) were performed with a cryostat (CM 1850 Leica Microsystems, Germany) according to the Kawamoto method (Kawamoto, 2003) and stained with hematoxylin and eosin. The mounting semi-permanent slides were examined under a microscope (OLYMPUS BX52 equipped with DP72 camera).

### Plant Growth and Quantitative Reverse Transcription PCR

The pathogen-related (PR) genes: *PR1a*, *PR1b*, *PR2*, *PR4*, *PBZ1*, or *PR10*, were identified as genes whose mRNA expression was induced as a response to the pathogen (van Loon, 1985). In rice, Yamamoto et al. (2010) and Chen et al. (2013, 2014) reported that mRNA expression of the PR genes was increased in plants showing symptoms of hybrid weakness and those genes demonstrated an autoimmune response. To confirm the autoimmune response of hybrid weakness in our study, mRNA expression of PR genes (*PBZ1*, *PR1a*, *PR4*, and *JIOsPR10*) were determined using 7-day-old seedlings grown at 24°C. Primers used for qRT-PCR are listed in Table 3. In summary, the F_2_ seeds which possessed severe weak genotype (*aabb*), weak genotype (*aaBb*, *Aabb)*, and normal genotype (*AaBB, AABb*), together with T65 and LTH were pre-germinated for 3 days. The germinated seeds were then sown on a mesh on the water in a growth chamber at 24°C. In this study, the electric heaters were used to control the water temperature. However, the air temperature was not controlled, but stable at approximately 22°C. Therefore, the temperature around seedlings was regarded to be at 24°C.

**TABLE 3:** List of primer sequences for qRT-PCR.

Gene	Primer sequence	References
Actin_f	TGTATGCCAGTGGTCGTACCA	Chen et al., 2013
Actin_r	CCAGCAAGGTCGAGACGAA	
PBZ1_f	TACACCATGAAGCTTAACCCTGCC	Chen et al., 2013
PBZ1_r	TCGAGCACATCCGACTTTAGGACA	
PR1a_f	GGTTATCCTGCTGCTTGCTGGT	Chen et al., 2013
PR1a_r	GTTGTGCGGGTCCACGAAGT	
PR4_f	AGTATGGATGGACCGCCTTCTGT	Chen et al., 2013
PR4_r	CTCGCAATTATTGTCGCACCTGTTC	
JIOsPR10_f	CCGGACGCTTACAACTAAATCG	Chen et al., 2013
JIOsPR10_r	CACTTCTCAATCACTGCTTGGAA	
		

The seedlings, leaf blades, and sheaths, were sampled 7 days after sowing, and the tip of the leaf blades was used for DNA extraction, and the remaining parts of the leaf blades and sheaths were used for total RNA extraction using the RNeasy plant mini kit (Qiagen Sciences, MD, United States) according to the manufacturer’s instructions. The RNA concentration was measured through QuantiFluor^®^ RNA System and Quantus fluorometer instrument (Promega, WI, United States). The ReverTra Ace^®^ qPCR RT Master Mix with gDNA Remover (TOYOBO, Osaka, Japan) was utilized to synthesize cDNA according to the manufacturer’s instructions using 500 ng total RNA. qRT-PCR was performed using StepOnePlus Real-Time PCR (Life Technologies, MD, United States).

## Results

### Segregation of Weakness

**FIGURE 2:**
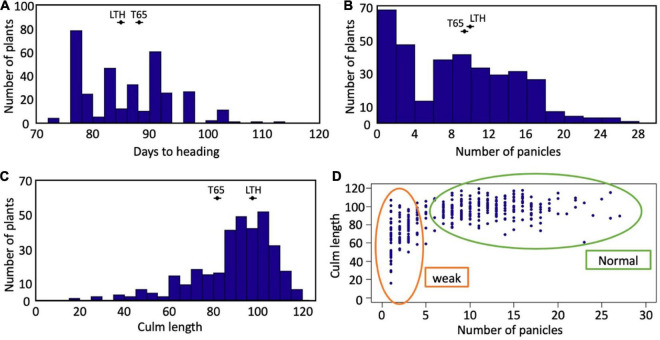
Segregation of traits in F_2_ population. Frequency distributions of **(A)** days to heading, **(B)** number of panicles, and **(C)** culm length. The averages of T65 and LTH were shown in each panel. **(D)** Scatter plot between number of panicles and culm length.

Segregation of weak plants was observed in the field condition (Supplementary Figure 1). The frequency distributions of days to heading, the number of panicles, and the culm length of the F_2_ population are shown in Figure 2. Segregation of these traits was continuous for days to heading and culm length (Figures 2A,C). A valley curve was observed in the number of panicles and can therefore be used to distinguish normal plants from weak plants (Figure 2B). In the correlation between the number of panicles and culm length (Figure 2D), the plants which had four or fewer panicles corresponded well to the weak plants which were visually discriminated.

### Quantitative Trait Loci Mapping

**FIGURE 3:**
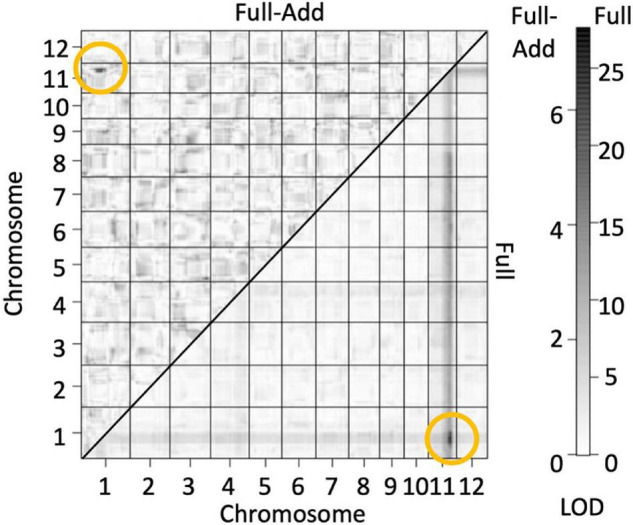
Heat map showing quantitative trait loci (QTL) for number of panicles by assuming interaction of two loci. Bottom and upper diagonals show the LOD score of full models (LOD_T_) and full-additive model (LOD_*Int*_ = LOD_T_ – LOD_x_ – LOD_y_), respectively. The yellow circles indicate sites with high LOD values and interaction between the two factors. The scale on the right shows LOD values of upper (left) and bottom (right) diagonals.

The genotypes of the 126 F_2_ plants from the population in 2016 were determined by GBS and a linkage map consisting of 862 single nucleotide polymorphism (SNP) markers were obtained. QTL analysis was performed using panicle number per plant as the indicator of hybrid weakness. We identified two significant QTL at a 5% level on chromosomes 1 and 11 (Table 4 and Supplementary Figure 2). Since the segregation of the weakness was controlled by these two major QTLs, each QTL was named *hybrid weakness j 1* (*hwj1*) (chromosome 1) and *hybrid weakness j 2* (*hwj2*) (chromosome 11). The interaction between *hwj1* and *hwj2* was detected by two-factor QTL analysis (Figure 3).

**TABLE 4:** Quantitative trait loci detected in F_2_ population.

QTL	Chr^a^	Trait	Position (Mb)	LOD	PVE (%)^b^	AE^c^	DE^d^
*hwj1*	1	Panicle number	18.53	4.76	15.97	1.53	–4.56
*hwj2*	11	Panicle number	22.37	12.78	37.32	–5.14	–0.59

*^a^Chromosome.*

*^b^Percentage of variance explained by the QTL.*

*^c^Additive effects of the marker calculated as (average of LTH-average of T65)/2.*

*^d^Dominance effect calculated as average of heterozygotes-(average of LTH + average of T65)/2.*

### Interaction of *hwj1* and *hwj2* Caused the Weakness

Since the hybrid weakness trait was associated with *hwj1* and *hwj2*, and the interaction between these two loci was significant (Figure 3 and Supplementary Figure 2), the genotypes of the 344 F_2_ plants were confirmed using the InDel markers linked to *hwj1* and *hwj2*. In this study, *hwj1* and *hwj2* loci were designated as *A* and *B*, wherein T65 and LTH genotypes were designated as *aaBB* and *AAbb*, respectively. The F_2_ plants carrying the genotypes of *Aabb* and *aaBb* showed weakness in the field. On the other hand, the double recessive genotype, *aabb*, showed a severe weak phenotype (Supplementary Figure 3).

### Phenotypic Characterization of Hybrid Weakness

**FIGURE 4:**
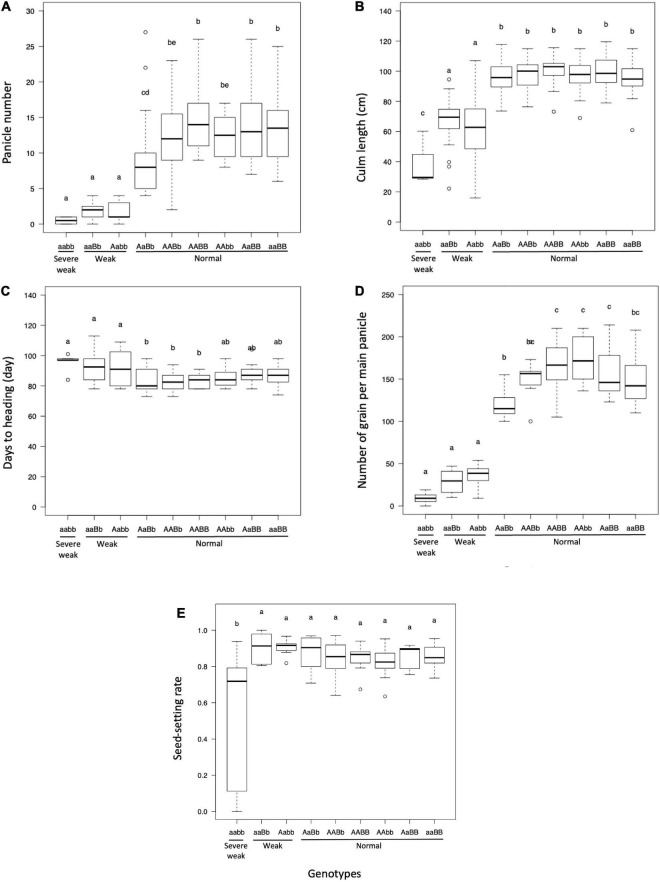
Boxplot of evaluated traits of F_2_ population in field condition: **(A)** panicle number, **(B)** culm length (cm), **(C)** days to heading, **(D)** number of grains per main panicle, and **(E)** seed-setting rate. Different letters in each phenotype represents significant differences among the genotypes based on one-way ANOVA followed by Tukey’s HSD test (*p* < 0.05).

The panicle number and culm length of plants with weak and severe weak genotypes were significantly reduced as compared to plants with normal genotypes (Figures 4A,B). The days to heading of the plants with weak and severe weak genotypes were delayed, but not significantly different from other genotypes in the field condition (Figure 4C). The number of grains per main panicle was significantly reduced in plants with weak and severe weak genotypes (Figure 4D). The seed setting rate of severe weak plants was significantly decreased compared to other genotypes (Figure 4E).

### Fine Mapping of the Causal Loci

**FIGURE 5:**
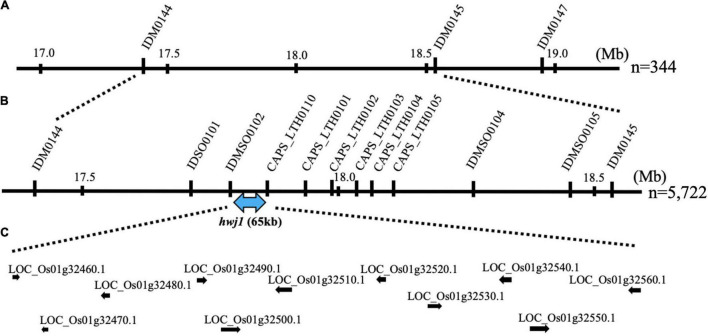
Fine mapping of the *hwj1* locus. **(A)** Initial mapping of *hwj1* on chromosome 1. Numbers show physical positions in megabase (Mb), **(B)** fine mapping of *hwj1*. The *hwj1* locus was delimited between flanking markers IDMSO0102 and CAPS_LTH0110. Blue-double head arrow shows candidate region, and **(C)** annotated genes in the candidate region. The black arrows represent the annotated genes.

**FIGURE 6:**
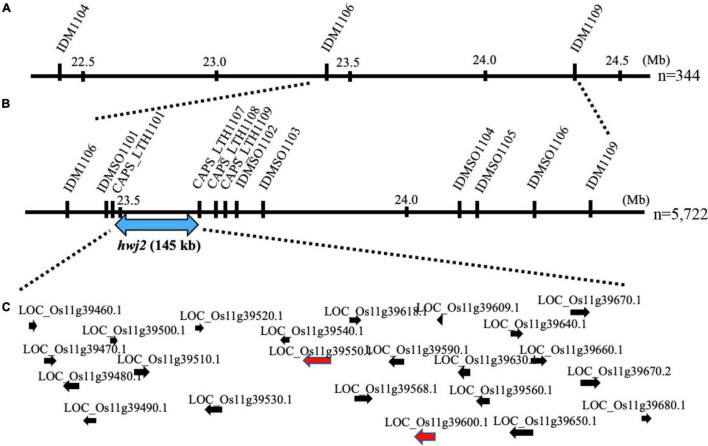
Fine mapping of the *hwj2* locus. **(A)** Initial mapping of *hwj2* on chromosome 11. Numbers show physical positions in megabase (Mb), **(B)** fine mapping of *hwj2*. The *hwj2* locus was delimited between flanking markers CAPS_LTH1101 and CAPS_LTH1107. Blue-double head arrow shows the candidate region, and **(C)** annotated genes in the candidate region. The red arrows represent NB-LRR disease-resistance gene and disease resistance-related gene. The black arrows represent other annotated genes.

To map the genes responsible for *hwj1* on chromosome 1 and *hwj2* on chromosome 11, larger F_2_ populations obtained from the T65 and LTH cross were grown in 2018 and 2019. A total of 5,722 plants were used for fine mapping, and the weakness phenotype was discriminated based on visual observation and the number of panicles. Using InDel and CAPS markers, the location of the identified causal loci were fine mapped. The *hwj1* locus was delimited to 65-kb by the flanking markers IDMSO_0102 and CAPS_LTH0110 on chromosome 1 (Figure 5). On the other hand, the location of the *hwj2* locus was delimited to 145-kb by the flanking markers CAPS_LTH1101 and CAPS_LTH1107 on chromosome 11 (Figure 6).

According to the annotation databases^2^,^3^, a total of eleven annotated genes were located in the region of *hwj1* (Figure 5 and Supplementary Table 3), and 22 annotated genes are located on the region of *hwj2* and includes one NB-LRR disease-resistance gene and one disease resistance-related gene containing NB-ARC domain (Figure 6 and Supplementary Table 4).

### Temperature Affects the Hybrid Weakness Caused by *hwj1* and *hwj2*

**FIGURE 7:**
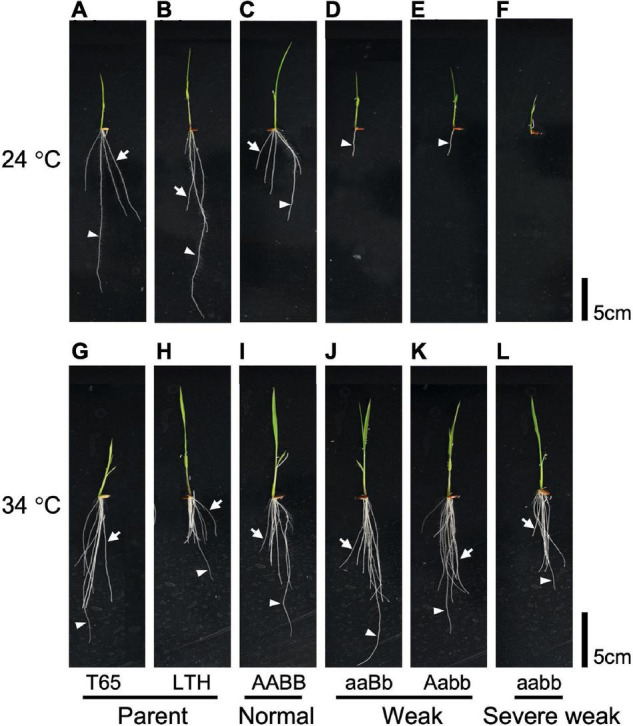
Morphology of 10-day-old seedlings grown under different temperatures. Seedlings grown at 24°C **(A)** T65, **(B)** LTH, **(C)** normal genotype, **(D,E)** weak genotypes, and **(F)** severe weak genotype; seedlings grown at 34°C **(G)** T65, **(H)** LTH, **(I)** normal genotype, **(J,K)** weak genotypes, and **(L)** severe weak genotype. Arrowheads point primary root. Arrows point crown root. Scale bars = 5 cm.

**FIGURE 8:**
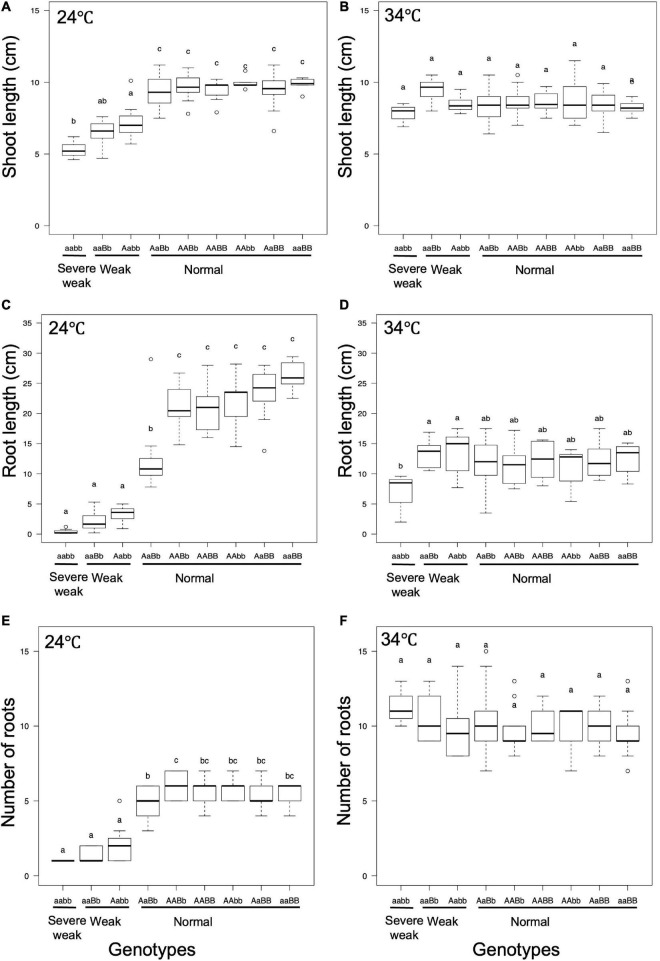
Boxplots of phenotypic evaluations of **(A,B)** shoot length, **(C,D)** root Length, and **(E,F)** number of roots of 10-day-old seedlings grown at 24 and 34°C. Different letters in each phenotype represent significant differences among the genotypes based on one way ANOVA followed by Tukey’s HSD test (*p* < 0.05).

To investigate the effect of temperature on the weak phenotype, the growth of the seedlings was examined at 24 and 34°C using 10-day-old seedlings. It was observed that 24°C enhances the weak phenotype and 34°C rescues the weakness (Figures 7, 8). At 24°C, the shoot length and the number of roots of severe weak genotypes (*aabb*) and weak genotypes (*aaBb*, *Aabb*) were significantly different from the normal genotypes (*AaBb, AABb, AABB, AAbb, AaBB*, and *aaBB*) (Figures 8A,E). In the same way, at 24°C, the root length of severe weak and weak genotypes was shorter compared to normal genotypes (Figure 8C). In addition, the root length of the F_1_ genotype (*AaBb*) was significantly different from other normal genotypes (Figure 8C). Similarly, the F_1_ genotype (*AaBb*) had fewer roots compared to other normal genotypes (Figure 8E). At 34°C, the shoot length, root length, and the number of roots showed no significant difference (Figures 8B,D,F), except for the root length of severe weak genotype (*aabb*) (Figure 8D).

### Morphological and Histological Observations of Embryo in Hybrid Weakness

**FIGURE 9:**
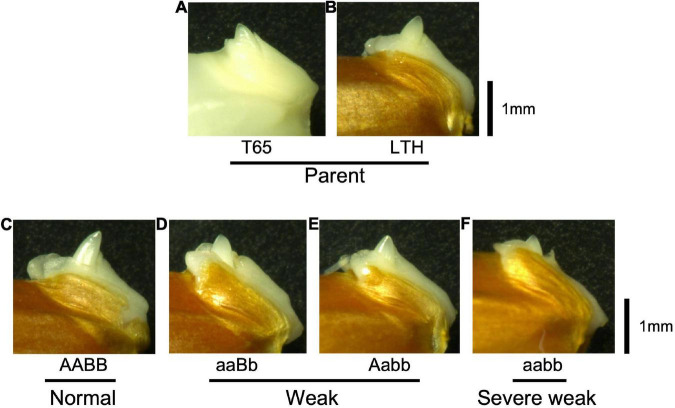
Morphology of embryos of F_2_ and the parents 1 day after soaking in water at 24°C. **(A)** T65, **(B)** LTH, **(C)** normal genotype, **(D,E)** weak genotypes, and **(F)** severe weak genotype. Scale bars = 1 mm.

**FIGURE 10:**
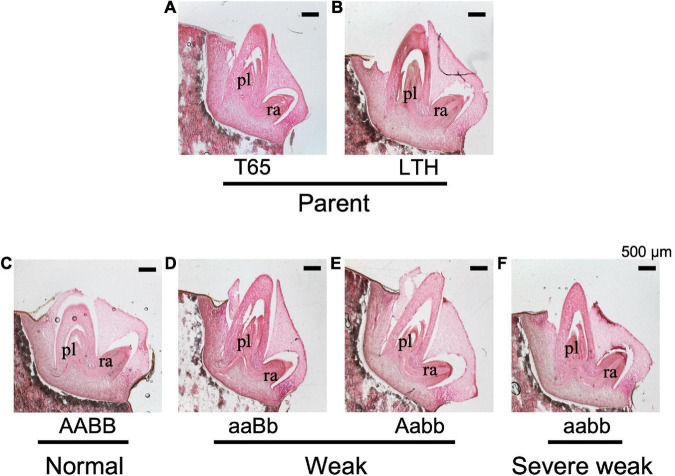
Histological characteristics of embryos of F_2_ and the parents in germinating seeds 1 day after soaking in water at 24°C. **(A)** T65, **(B)** LTH, **(C)** normal genotype, **(D,E)** weak genotypes, and **(F)** severe weak genotype. The sections (20 μm thick) were stained with hematoxylin and eosin. pl, plumule (shoot meristem); ra, radicle. Scale bars = 500 μm.

To confirm whether an abnormality occurred in the embryo, morphological and histological analyses on 1-day-soaked embryos at 24°C were conducted. The seeds were genotyped before soaking using the half seeds. All the F_2_ genotypes and parents showed no visible morphological differences (Figure 9). In addition, structures of the shoot meristems and radicles observed by vertical sections were normal in weak and severe weak genotypes (Figures 10D–F), compared to those of normal genotypes and the parents (Figures 10A–C).

### Quantitative Reverse Transcription PCR of Pathogenesis-Related Genes

**FIGURE 11:**
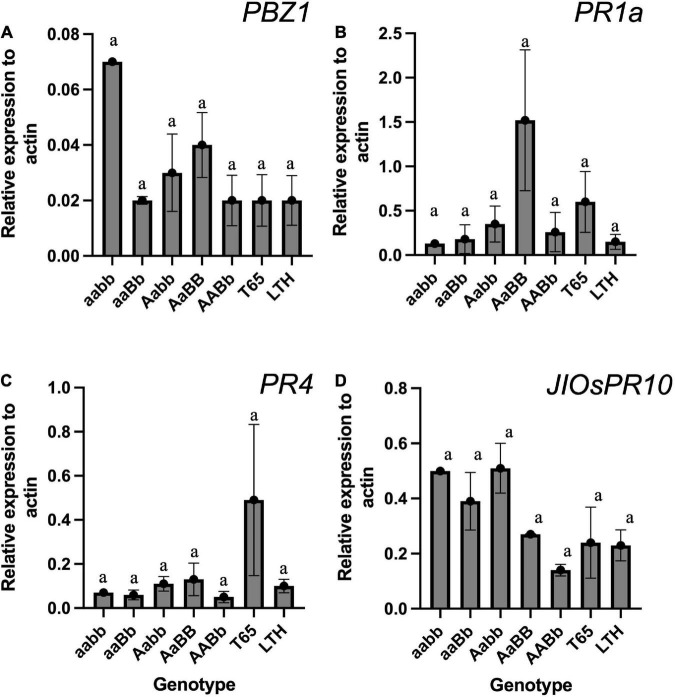
The expression level of pathogen-related genes (PRs) in the leaf blades and sheaths of severe weak genotype (*aabb*), weak genotypes (*aaBb* and *Aabb*), normal genotype (*AaBB* and *AABb*), and the parents (T65 and LTH) at 7-day-old seedling stage. **(A)** *PBZ1*, **(B)** *PR1a*, **(C)** *PR4*, and **(D)** *JIOsPR1*. Each qRT-PCR analysis was performed with three biological replications, but severe weak plants had no replication. Bars represent the means ± SE. Actin was used as the endogenous control for normalization.

To confirm the autoimmune response that was often detected in previous studies on hybrid weakness, the mRNA expressions of PR genes were compared among genotypes using the seedlings grown at 24°C. The result of qRT-PCR did not show any significant difference in the mRNA expression when compared across genotypes (Figure 11). However, the *PBZ1* showed a tendency of increased mRNA levels in severe weak genotypes (Figure 11A). The same tendency was observed in *JIOsPR10* of weak and severe weak genotypes (Figure 11D).

## Discussion

In the present study, the segregation of weak plants was observed in the F_2_ population derived from the cross between T65 and LTH. Generally, the symptoms of hybrid weakness include short stature, less tiller number, impaired root development, late heading date, and significantly decreased grain yield. All these phenomena were observed in weak and severe weak plants (Figure 4) under field conditions. The primary cause of weakness was the suppression of crown root formation at a relatively low temperature and the phenotypes of plants with weak and severe weak genotypes were rescued at high temperatures (Figure 7). In contrast to the clear difference observed in the seedling test at 24°C (Figure 7), continuous segregation was observed in the field (Figure 2). This may be due to the temperature in the field which is between 24 and 34°C, and the expression of the weak phenotype was not stable. However, root systems of the weak and severe weak plants in field condition were smaller than the normal plants (data not shown).

Through GBS and QTL analysis, it was indicated that the hybrid weakness was caused by the interaction of two major QTLs. The hybrid weakness explained by the interaction of two genes has been reported by many scientists (e.g., Oka, 1957; Kubo and Yoshimura, 2002; Yamamoto et al., 2007, 2010; Chen et al., 2014). The two loci in the present study were designated as *hwj1* (chromosome 1) and *hwj2* (chromosome 11). The genotype of T65 and LTH was assumed to be *hwj1hwj1Hwj2Hwj2* (*aaBB*) and *Hwj1Hwj1hwj2hwj2* (*AAbb*), respectively. The plants possessing 3 recessive alleles (*aaBb* and *Aabb*) showed a weak phenotype, and the *aabb* genotype resulted in severe weakness, thus the expected segregation ratio of normal: weak: severe weak plants is 11: 4: 1 (Supplementary Figure 3). This model of inheritance is similar to that of Fukuoka et al. (2005), although most of the previous studies reported a segregation ratio of normal: weak = 15:1.

Because of the interaction of the two loci and the unstable phenotype in the field, fine mapping of the two loci was done using plants with clear phenotypes. A total of 5,722 F_2_ plants were used for fine mapping, and the region of *hwj1* and *hwj2* were narrowed down to 65-kb on chromosome 1 (Figure 5) and 145-kb on chromosome 11 (Figure 6), respectively. In the 65-kb candidate region of *hwj1*, 11 genes were predicted, and some of these are retrotransposon-like genes (Nadir et al., 2019). On the other hand, the candidate region of *hwj2* contains two disease resistance-related genes, thus these two genes were primary candidates for *hwj2*. The sequencing of the candidate regions and transgenic experiments are needed to confirm the causal genes.

In general, several hybrid weakness cases in some plants were reported to be low temperature-dependent and phenotypes of weak plants were rescued at high temperature (Saito et al., 2007; Alcázar et al., 2009; Shiragaki et al., 2021; Yoneya et al., 2021). To date, only one high temperature-dependent hybrid weakness in rice was reported (Chen et al., 2014). In our study, it was observed that the hybrid weakness was induced by low temperature (24°C), and weakness syndrome did not appear at high temperature (34°C). At 24°C, the shoot length, root length, and the number of roots of 10-day-old weak and severe weak genotypes were significantly shorter compared to normal genotypes (Figures 8A,C,E). Moreover, the root length of severe weak genotypes (*aabb*) was shorter than 1 cm (Figure 8C) and they did not grow crown roots (Figure 7F). This morphological feature of severe weak phenotype is the same as the previous description by Ichitani et al. (2007) wherein plants that were homozygotes for *Hwc1* and *Hwc2* showed a very short shoot and did not grow roots. In our study, the root length of weak genotypes (*aaBb*, *Aabb*) was shorter than 5 cm with few crown roots (Figures 8C,E). Furthermore, the root length and number of roots of the F_1_ genotype (*AaBb*) were different from other normal genotypes (*AABb, AABB, AAbb, AaBB*, and *aaBB*) (Figures 8C,E). Conversely, at 34°C, the shoot length, root length, and number of roots of the severe weak and weak genotypes were restored. To date, several studies were also reported wherein plants with weak genotypes were rescued in high temperatures such as in *Nicotiana* species (Yamada et al., 1999) and *Gossypium* species (Philips, 1977). Thus, high temperature treatment might be generally effective in rescuing hybrid plants from weakness. Taken together, hybrid weakness in rice affects more the root than the shoot.

According to morphological and histological observations, the embryos of weak and severe weak genotypes were normal (Figure 9). Taken together, hybrid weakness simply affects postembryonic development and is independent of embryogenesis. In addition, hybrid weakness is triggered by low temperature after germination. In rice, the temperature sensitivity was reported by the previous studies such as *Hwc* genes (Saito et al., 2007), *hw3/hw4* genes (Fu et al., 2013), *thb1* (Yoneya et al., 2021) where low temperature enhanced the weakness, while *Hwi* genes (Chen et al., 2013) enhanced the weakness at high temperature.

In our study, the candidate region of *hwj2* contained disease resistance-related genes. So far, at least one of the causal gene combinations for rice hybrid weakness was disease-related genes (e.g., Yamamoto et al., 2010; Chen et al., 2014). Therefore, it was hypothesized that of *hwj1* and *hwj2* were disease-related genes. According to the annotation databases (see text footnote 2 and 3), one LRR (leucine-rich repeat) family protein (LOC_Os11g39550) and one disease resistance-related gene containing NB-ARC domain (LOC_Os11g39600) is located in the candidate region of *hwj2*, implying that these genes are the causal genes. However, many other genes are also located in that region (Supplementary Table 4). The *Hwa1* and *Hwa2* (Ichitani et al., 2011) and *hwg2* (Fukuoka et al., 2005) shared the same candidate region as *hwj2* on chromosome 11. Thus, there is a possibility that a common allele of an NB-LRR gene is the cause of hybrid weakness observed in different crosses and different counterpart genes.

In previous studies, the mRNA expression levels of the PR genes were upregulated in plants showing symptoms of hybrid weakness (Yamamoto et al., 2010; Chen et al., 2014). Contrary to the expectation, the results of this study did not show a significant increase in the mRNA levels of PR genes (Figure 11). However, a tendency of autoimmune response was observed in *PBZ1* of severe weak genotype (Figure 11A), and the same tendency was found in *JIOsPR10* of both weak and severe weak genotypes (Figure 11D). Thus, the authors presume that the hybrid weakness is accompanied by temperature sensitivity and is caused by an autoimmune response. This result should be carefully translated because RNA was extracted from the aerial part of young seedlings where no symptom was observed. To date, several studies have shown that the activation of PR genes is associated with the occurrence of symptoms under various types of conditions (Yamamoto et al., 2010; Chen et al., 2014; Shiragaki et al., 2019). Therefore, sampling factors such as growth stage and organ source must be taken into consideration.

## Conclusion

In the present study, the phenotypic property of the F_2_ weakness observed in the T65/LTH cross was characterized and two interacting loci, *hwj1* and *hwj2*, were identified. These loci were identified to be associated with temperature-sensitive hybrid weakness. To clarify the molecular mechanism of the hybrid weakness, cloning of the *hwj1* and *hwj2* by further sequencing and transgenic experiments is necessary. In addition, it is also necessary to clarify the mRNA expression of PR genes to confirm if the hybrid weakness was caused through the mechanism of an autoimmune response. The distribution of *hwj1* and *hwj2* alleles is also of future interest.

## Data Availability Statement

The datasets presented in this study can be found in online repositories. The names of the repository/repositories and accession number(s) can be found below: https://www.ddbj.nig.ac.jp/, DRA014130.

## Author Contributions

HS and KD: conceptualization and data curation. TKS, MK, HS, YI, and KD: investigation. TKS and HS: writing—original draft preparation. TKS, VPR, SN, and KD: writing—review and editing. KD: supervision. YI and KD: funding acquisition. All authors read and approved the submitted version.

## Conflict of Interest

HS was employed by Environmental Control Center Co., Ltd. The remaining authors declare that the research was conducted in the absence of any commercial or financial relationships that could be construed as a potential conflict of interest.

## Publisher’s Note

All claims expressed in this article are solely those of the authors and do not necessarily represent those of their affiliated organizations, or those of the publisher, the editors and the reviewers. Any product that may be evaluated in this article, or claim that may be made by its manufacturer, is not guaranteed or endorsed by the publisher.

## Footnotes

1
http://r-project.org/

2
http://rice.plantbiology.msu.edu/

3
http://rapdb.dna.affrc.go.jp/
