# Supplementary material for: A Novel Combination of Genes Causing Temperature-Sensitive Hybrid Weakness in Rice
Source: Front Plant Sci. 2022 Jun 28;13:908000. doi: 10.3389/fpls.2022.908000 (PMC9274174; doi:10.3389/fpls.2022.908000)
Supplement: Supplementary file 1 [file Data_Sheet_1.PDF]

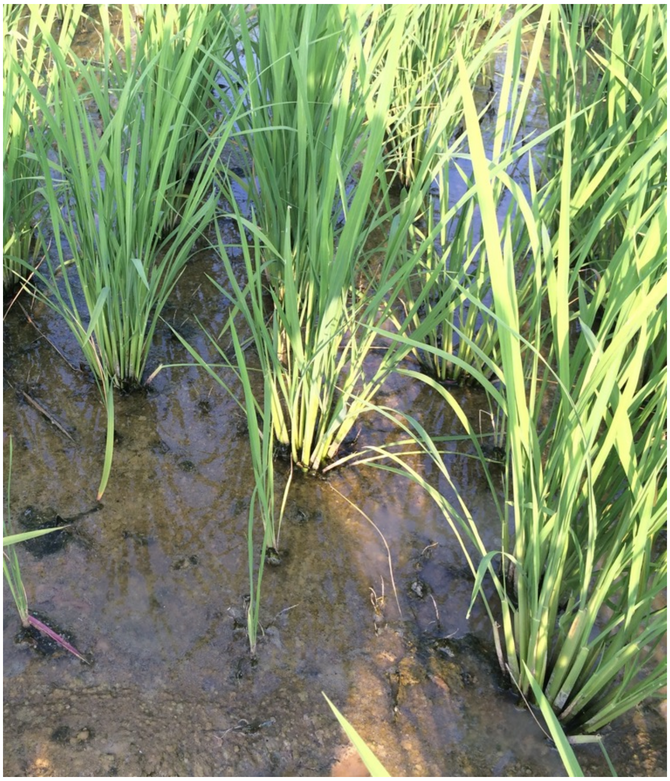

Supplementary Figure 1. Segregation of weak plants in T65/LTH F_2 population grown in the field.

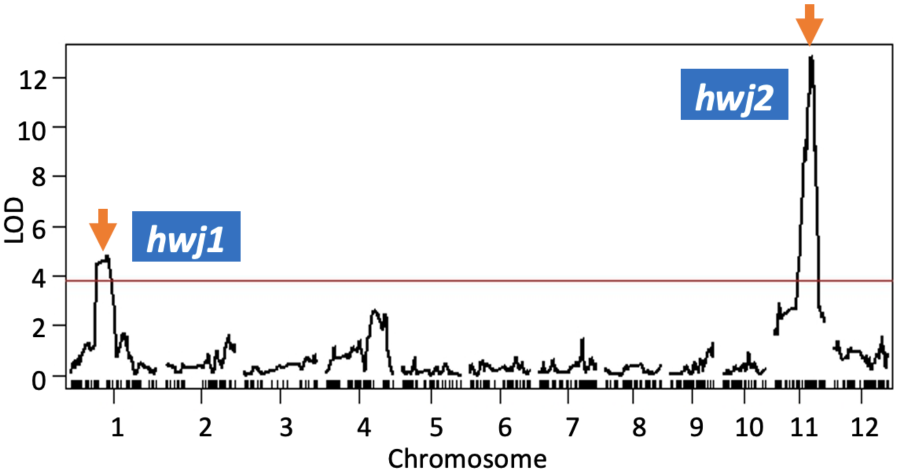

Supplementary Figure 2. QTL mapping using number of panicles of the 126 individuals in T65/LTH F_2 population. Red line represents significance threshold at 5% level. The arrows indicate significant QTLs at 5% level. Tick marks on the horizontal axis indicate marker positions.

T65 = *hwj1hwj1 Hwj2Hwj2* = aaBB

LTH = *Hwj1Hwj1 hwj2hwj2* = AAbb

	AB	Ab	aB	ab
AB	AABB	AABb	AaBB	AaBb
Ab	AABb	AAbb	AaBb	Aabb
aB	AaBB	AaBb	aaBB	aaBb
ab	AaBb	Aabb	aaBb	aabb

Normal : Weak : Severe weak

11 : 4 : 1

Supplementary Figure 3. A punnett square showing the inheritance of weakness observed in F_2 population in the field. When *hwj1* and *hwj2* loci were designated as A and B, T65 and LTH possessed aaBB and AAbb genotypes, respectively. The white cells indicate normal genotypes. The light blue cells indicate weak genotypes. The dark blue cell indicates severe weak genotype.

Supplementary Table 1. List of CAPS markers for fine mapping of *hwj1* locus

No.	Marker	Primer Sequence	Position	Enzyme
1	CAPS_LTH0101f	aggcctgtttgagctgtcca	17,937,841	NlaIII
	CAPS_LTH0101r	tcaacaacccgctgctttct		
2	CAPS_LTH0102f	ttaggcgtgcccatgctaat	17,994,314	NlaIII
	CAPS_LTH0102r	gcactgctgtctgctcctga		
3	CAPS_LTH0103f	cagtgggcaacgagaagacc	18,032,911	TaqI
	CAPS_LTH0103r	ccgtcgaaggagtcgatttg		
4	CAPS_LTH0104f	agcaggttgtccctccatga	18,062,841	MspI
	CAPS_LTH0104r	tgagggataaggtgcgcagt		
5	CAPS_LTH0105f	cggcacatgaagcagctaga	18,105,431	NlaIII
	CAPS_LTH0105r	tttctgcgcctgtggttctt		
6	CAPS_LTH0110f	ctaaagacccctcccccaag	17,862,744	HpyCH4V
	CAPS_LTH0110r	gcagctctcccttggacctt		

Supplementary Table 2. List of CAPS markers for fine mapping of *hwj2* locus

No.	Marker	Primer Sequence	Position	Enzyme
1	CAPS_LTH1101f	tgcatcgcaggaagcagata	23,494,766	AfaI
	CAPS_LTH1101r	ccccagctgccatgtaactc		
2	CAPS_LTH1107f	gacatgcaagtcatgccaca	23,639,840	TaqI
	CAPS_LTH1107r	tggattcgttcatgggtctc		
3	CAPS_LTH1108f	cttttgcggtgtggttgatg	23,666,836	AfaI
	CAPS_LTH1108r	gctgcaaatcgtccaagacc		
4	CAPS_LTH1109f	gacggtggtcgctgacttct	23,670,478	HhaI
	CAPS_LTH1109r	cggagggctcttgaaaacct		

Supplementary Table 3. List of annotated genes on the region of *hwj1* according to the annotation databases (http://rapdb.dna.affrc.go.jp/ and http://rice.plantbiology.msu.edu/)

**Gene ID**	**Description**	**Position**
LOC_Os01g32460.1	expressed protein	17,798,261-17,799,032
LOC_Os01g32470.1	retrotransposon protein, putative, unclassified	17,803,151-17,804,029
LOC_Os01g32480.1	retrotransposon, putative, centromere-specific	17,808,859-17,809,254
LOC_Os01g32490.1	hypothetical protein	17,817,775-17,818,718
LOC_Os01g32500.1	retrotransposon protein, putative, unclassified, expressed	17,820,210-17,826,419
LOC_Os01g32510.1	retrotransposon protein, putative, unclassified, expressed	17,827,164-17,831,627
LOC_Os01g32520.1	expressed protein	17,836,906-17,837,466
LOC_Os01g32530.1	transposon protein, putative, CACTA, En/Spm sub-class	17,840,970-17,843,733
LOC_Os01g32540.1	acanthoscurrin-2 precursor, putative, expressed	17,846,238-17,846,534
LOC_Os01g32550.1	hypothetical protein	17,846,809-17,849,850
LOC_Os01g32560.1	expressed protein	17,859,771-17,860,643

Supplementary Table 4. List of annotated genes on the region of *hwj2* according to the annotation databases (http://rapdb.dna.affrc.go.jp/ and http://rice.plantbiology.msu.edu/)

Gene ID	Description	Position
LOC_Os11g39460.1	retrotransposon protein, putative, unclassified, expressed	23,499,869-23,501,852
LOC_Os11g39470.1	retrotransposon protein, putative, unclassified	23,502,377-23,504,915
LOC_Os11g39480.1	jacalin-like lectin domain containing protein, expressed	23,506,616-23,507,170
LOC_Os11g39490.1	jacalin-like lectin domain containing protein, expressed	23,508,365-23,513,127
LOC_Os11g39500.1	hypothetical protein	23,514,275-23,514,644
LOC_Os11g39510.1	retrotransposon protein, putative, unclassified, expressed	23,522,471-23,530,564
LOC_Os11g39520.1	hypothetical protein	23,535,118-23,536,097
LOC_Os11g39530.1	jacalin-like lectin domain containing protein, expressed	23,537,272-23,545,497
LOC_Os11g39540.1	14-3-3 protein, putative, expressed	23,553,560-23,558,464
LOC_Os11g39550.1	Leucine Rich Repeat family protein, expressed	23,565,236-23,575,489
LOC_Os11g39568.1	expressed protein	23,578,838-23,581,592
LOC_Os11g39580.1	expressed protein	23,601,796-23,604,296
LOC_Os11g39590.1	expressed protein	23,585,625-23,590,396
LOC_Os11g39600.1	resistance protein, putative, expressed	23,592,859-23,595,271
LOC_Os11g39609.1	OsFBDUF64 - F-box and DUF domain containing protein	23,596,922-23,598,259
LOC_Os11g39618.1	expressed protein	23,578,838-23,581,592
LOC_Os11g39630.1	ZOS11-08 - C2H2 zinc finger protein, expressed	23,600,980-23,603,659
LOC_Os11g39640.1	zinc finger, C3HC4 type domain containing protein, expressed	23,610,858-23,612,864
LOC_Os11g39650.1	WD domain, G-beta repeat domain containing protein, expressed	23,615,037-23,619,315
LOC_Os11g39660.1	transposon protein, putative, Ac/Ds sub-class, expressed	23,623,013-23,625,543
LOC_Os11g39670.1	seryl-tRNA synthetase, putative, expressed	23,628,182-23,632,588
LOC_Os11g39680.1	expressed protein	23,635,337-23,636,133
